# The age-gender-status profile of high performing athletes in the UK taking nutritional supplements: Lessons for the future

**DOI:** 10.1186/1550-2783-5-2

**Published:** 2008-01-10

**Authors:** Andrea Petroczi, Declan P Naughton

**Affiliations:** 1 Kingston University, School of Life Sciences, Faculty of Science, Kingston upon Thames, Surrey, KT1 2EE, UK

## Abstract

**Background:**

Owing to the mechanics of anti-doping regulation via the World Anti-Doping Agency's Prohibited List, nutritional supplement use received little attention in comparison to the prevalence of doping. The aims of this study were to investigate supplement use, identify groups of athletes with high levels of supplement use and the prevalence of concomitant use of supplements.

**Methods:**

Survey data from 847 high-performing athletes in the UK were analysed using descriptive statistics. The survey, conducted by UK Sport, consisted of questions regarding knowledge of the prohibited substances, testing procedure, nutritional supplement use and perceptions of the doping problem. The proportion of supplement users and the relative use of each supplement were compared by age, gender and professional status.

**Results:**

Among 874 high-performing athletes in the UK sample, 58.8% of them reported the use of at least one nutritional supplement. Among supplement users, 82.6% used more than one and 11.5% reported use of more than five nutritional supplements. Of the 9 supplements listed, multivitamins (72.6%) and vitamin C (70.7%) were used most, followed by creatine (36.1%), whey protein (31.7%), echinacea (30.9%), iron (29.9%) and caffeine (23.7%). Less than 11% reported the use of magnesium or ginseng. Creatine use was typically associated with males regardless of status and across all ages, whereas iron was characteristically used by females. A 'typical' supplement user is male, between 24 and 29 years of age, involved in professional sport and using a combination of supplements. Male professional players between age 30 and 34 years, and female non-professional athletes between 24 and 29 years of age also represented a considerable proportion of supplement users. Athletes older than 40 years of age were practically non-users. Concomitant use of supplements is characteristic of male users more than females.

**Conclusion:**

As supplement use has been previously shown to increase the probability of prohibited substance use, groups exhibiting high use of nutritional supplements should be monitored. Future research should incorporate a wide range of supplements and enquire about the daily amount ingested. In addition to tutoring, preventive measures should incorporate offering acceptable and healthy alternatives for assisted performance enhancement.

## Background

Owing to the mechanics of the anti-doping regulation via the World Anti-Doping Agency's Prohibited List, nutritional supplement use has received little attention in the past in comparison to the prevalence of doping despite a growing body of evidence, which supports the assumption that performance enhancing supplement use positively correlates with the probability of prohibited performance enhancing substance use [1-3]. Supplement taking has also been used as a proxy for attitude toward prohibited substances [4]. A noticeable positive association was found between supplement use and i) a perception that doping is a problem and ii) knowledge of doping testing procedure. The interpretation of such findings may be that supplement users have the need or desire to assist their performance but wish to do so by legal means, or alternatively, supplement use is the first step in their 'pharmacological training' [5].

Another reason that underscores the importance of research into supplement use is the pressure for constant enhancement of sport performance and the need for control of performance enhancement. In the UK, the Science and Technology Committee of the UK Parliament House of Commons conducted an extensive investigation into human performance enhancement in sport [6]. Among the recommendations, all stakeholders were urged to increase their research effort aiming to find acceptable legal alternatives for performance enhancements. One of these legal alternatives is the confidence in nutritional supplements with proven ergogenic effects. However, certain nutritional supplements can also be detrimental to health; hence their use should be closely monitored.

Vitamins A (retinol), D, E, and K are stored in the liver and in body fat stores hence they do not need to be taken every day. Some are toxic if an excess amount is consumed. For example, evidence was found that beta-carotene supplements and retinol supplements may increase the risk of lung cancer and there is suggestive evidence that links selenium to skin cancer [7]. In a recent meta-analysis of primary and secondary treatment with antioxidant supplements, beta-carotene, vitamin A and E were also found to increase mortality but there was no convincing evidence for harmful effects of selenium or vitamin C [8]. Despite the fact that water-soluble vitamins (i.e. C, B) are excreted in the urine rather than stored in the body, there is evidence suggesting that vitamin C, especially in combination with iron [9] can cause damage to the gastrointestinal tract (GI) and initiate or aggravate symptoms associated with chronic GI disorders. A high intake of iron, especially in combination with manganese doubles the risk for Parkinson's disease [10]. Unnecessary cobalt ingestion results in enhanced oxidative stress leading to organ damage and dysfunction in liver and kidney, and also impairs thyroid activity and myocardial function [11]. In addition to the potential hazard of positive doping tests [12,13], contamination may also pose health risks such as lead contamination in calcium products [14].

In this study, we provide a nutritional supplement users' profile of UK high-performance athletes to inform policy makers and researchers. The aims of this report were to: i) describe the profile of supplement users by age, gender and professional status and ii) describe the prevalence of supplement use and multiple supplement use among high-performing athletes.

## Methods

### Data

Data were collected by the UK Sport for the 2005 Drug Free Sport [15] report among high performing athletes in the UK, aged 18 years and over. At the start of the project, 2995 surveys were sent to randomly selected athletes via the respective Sports councils or Professional bodies. As the participation was voluntary with no selection criteria, all athletes had an equal chance of being included in the sample. The survey data of 874 athletes were re-analysed to identify athlete groups with extensive supplement use. In the overall sample European football was represented with 17.4%, followed by badminton (14.7%), athletics (11.3%), cricket (7.2%), rugby (6.6%), swimming (6.4%) and cycling (6.2%). The remaining 36.7% comprised 30 different sports [15], each contributing less than 3.5%. Professional athletes (n = 272) were from football (52.9%), cricket (12.5%), rugby union (21.3%), tennis (5.5%), ice hockey (4.1%) and basketball (3.7%). Non-professionals were lottery-funded athletes from 34 sports (some with disability variations). Sports represented in the full and supplement user samples are listed in Table 1.

**Table 1:** The distribution of lottery funded athletes in the full and supplement user samples by sports and gender

**Sport**	**Full sample n = 874 (100%)**		**Supplement users n = 520 (100%)**	
	**Male**	**Female**	**Male**	**Female**

Archery	4 (< 1%)	2 (< 1%)	1 (< 1%)	1 (< 1%)
Athletics ^a^	53 (6.1%)	44 (5.0%)	26 (5.0%)	35 (6.7%)
Badminton	30 (3.4%)	34 (3.9%)	8 (1.5%)	18 (3.5%)
Boccia	1 (< 1%)	0	0	0
Boxing	10 (1.1%)	0	4 (< 1%)	0
Canoeing	16 (1.8%)	8 (< 1%)	9 (1.7%)	8 (1.5%)
Cricket	14 (1.6%)	15 (1.7%)	3 (< 1%)	7 (1.4%)
Cycling	39 (4.5%)	19 (3.6%)	29 (5.6%)	16 (3.1%)
Diving	1 (< 1%)	3 (< 1%)	0	3 (< 1%)
Equestrian	0	7 (< 1%)	0	1 (< 1%)
Fencing ^b^	1 (< 1%)	0	0	0
Football (European)	0	8 (< 1%)	0	5 (1.0%)
Golf	9 (1.0%)	11 (1.2%)	1 (< 1%)	5 (1.0%)
Gymnastics	12 (1.3%)	8 (< 1%)	5 (1.0%)	3 (< 1%)
Hockey	11 (1.2%)	7 (< 1%)	5 (1.0%)	4 (< 1%)
Ice skating	1 (< 1%)	3 (< 1%)	1 (< 1%)	1 (< 1%)
Judo	4 (< 1%)	7 (< 1%)	4 (< 1%)	4 (< 1%)
Karate	1 (< 1%)	1 (< 1%)	1 (< 1%)	0
Modern Pentathlon	2 (< 1%)	8 (< 1%)	1 (< 1%)	6 (1.1%)
Netball	0	8 (< 1%)	0	2 (< 1%)
Powerlifting	1 (< 1%)	1 (< 1%)	0	0
Rowing	14 (1.6%)	10 (1.1%)	12 (2.3%)	8 (1.5%)
Sailing	9 (1.0%)	6 (< 1%)	4 (< 1%)	0
Shooting ^a^	10 (1.1%)	9 (1.0%)	3 (< 1%)	1 (< 1%)
Squash	8 (< 1%)	5 (< 1%)	5 (1.0%)	5 (1.0%)
Swimming ^a^	27 (3.1%)	29 (3.3%)	17 (3.3%)	19 (3.6%)
Table tennis ^a^	3 (< 1%)	5 (< 1%)	0	4 (< 1%)
Taekwondo	0	2 (< 1%)	0	1 (< 1%)
Triathlon	1 (< 1%)	7 (< 1%)	0	6 (1.1%)
Tennis ^a^	0	3 (< 1%)	0	2 (< 1%)
Waterskiing	2 (< 1%)	0	0	0
Wheelchair basketball ^b^	54 (6.2%)	1 (< 1%)	2 (< 1%)	1 (< 1%)
Wheelchair rugby ^b^	5 (< 1%)	1 (< 1%)	3 (< 1%)	1 (< 1%)
Wheelchair tennis ^b^	2 (< 1%)	0	1 (< 1%)	1 (< 1%)
Missing data on sport	285 (32.6%)	15 (1.7%)	193 (37.1%)	8 (1.5%)
Missing data on gender	5 (< 1%)			

Total	580 (66.4%)	289 (33.1%)	338 (65.0%)	182 (35.0%)

Percentages to full sample and supplement user sample are in parentheses.

^a ^Includes disability sport.

^b ^Disability sport only

### Statistical analyses

Results are presented as descriptive statistics summarised in tables and charts. For the relative use of each supplement by athlete groups, the number of users of a given supplement was divided by the total number of supplement users in the respective age-gender-status group. The use of each supplement per sub-group is shown as a proportion of the 100% for the given supplement. Chi-square goodness of fit statistics test was used to establish significant differences between the proportion of each age-gender-status group in the full sample and among supplement users. Chi-square statistics test was performed under the assumption that the proportion of each subgroup in the supplement user sample does not differ from the proportion of each subgroup observed in the random sample (H_0_: expected frequency = observed frequency). If sufficient evidence is found to reject the null hypothesis, it suggests that the proportion of a given subgroup among supplement users in the population is higher or lower than it could be expected by random chance. The statistically significant difference, however, does not necessarily manifest in a large difference in the sample.

## Results

Of the 2995 questionnaires distributed, 874 were returned and 520 met the criteria for further analysis for supplement use (346 indicated no use of nutritional supplements and 8 were excluded because of missing data).

**Figure 1:**
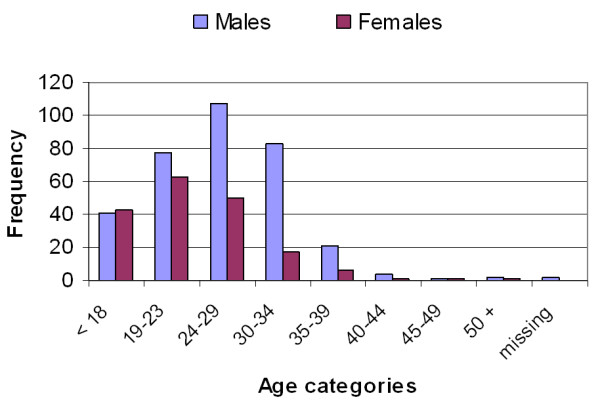
Age distribution of male and female supplement user athletes (n = 520).

The supplement user athlete sample comprised of 191 professional (186 male and 5 female) and 329 lottery-funded (152 male and 177 female) athletes from 31 sports (Table 1). The most dominant age group among male supplement user athletes was the 24 – 29 years of age group (31.6%), whereas the largest proportion (34.6%) of females supplement users was the 19 – 23 years age group (Figure 1).

Among the supplements, the use of multivitamins and vitamin C were reported by 72.6% and 70.7%, respectively. Creatine was used by 36.1%, followed by whey protein (31.7%), echinacea (30.9%), iron (29.9%), caffeine (23.7%) and magnesium or ginseng with less than 11%.

### User profile

**Figure 2:**
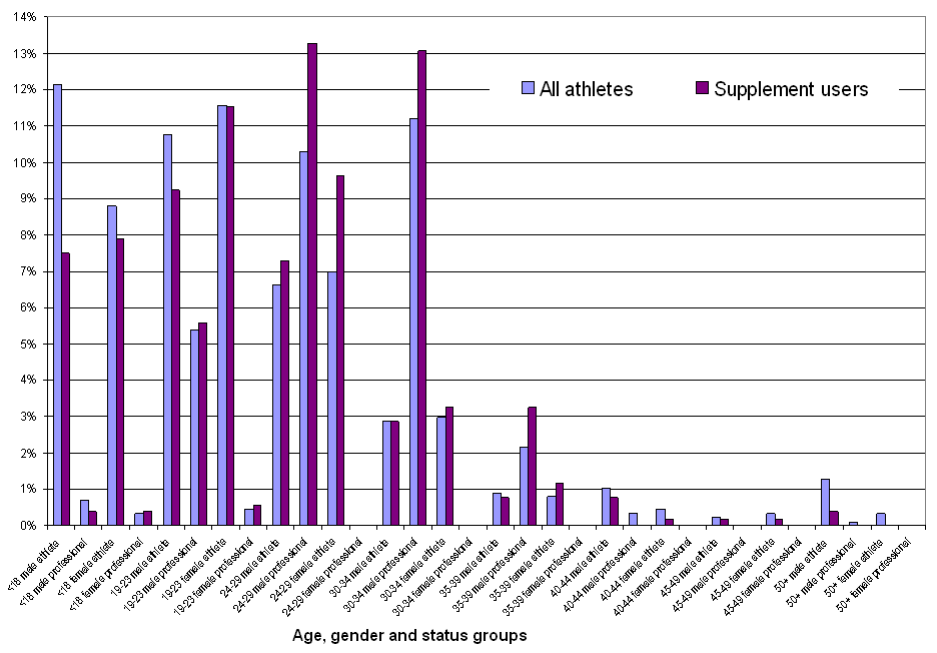
Comparison of relative percentage (Y-axis) of athlete subgroups in the full sample (n = 874) and supplement user sample (n = 520).

Overall, the supplement user sample (n = 520) did not differ significantly from the entire cohort, but for three sub-groups (Figure 2). The supplement user proportion of the 24–29 years old male professional athletes who reported supplement use was increased by 3% (χ^2 ^= 4.46, p = .035), from 10% (proportion of the 24–29 years old professional male athletes in the total sample) to 13% (proportion of the 24–29 years old professional male athletes who reported supplement use in the total supplement user sample). Similarly, the proportion of the 24–29 years old female lottery funded athletes has increased from 7% to 10% (χ^2 ^= 5.18, p = .023) in the supplement user cohort relative to the full sample. The proportion of the under-18 years old male lottery-funded athletes slightly decreased from 12% in the full sample to 8% in the supplement user cohort (χ^2 ^= 9.18, p = .002). Although the change in the sample appears to be small (3 – 4%), it suggests that the characteristics of the identified groups (a combination of gender, age and status) related to supplement use in the athlete population. Athletes over 40 years of age represented 4.1% of the athletes in the full sample and only 1.7% in the supplement user sample (Figures 1 and 2).

The most dominant group among supplement users appears to be the male, professional in the 24–34 years age group (Figure 2). This resonates with the literature where male athletes were found to be more likely to use performance enhancements [2,3,16-18]. Observed supplement use was also comparably high among female athletes between age 24 and 29 years (Figure 2).

**Figure 3:**
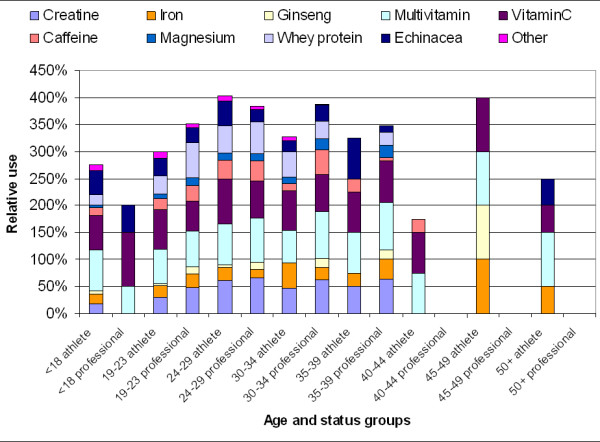
Relative use of nutritional supplements by age and status among male supplement users (n = 520).

**Figure 4:**
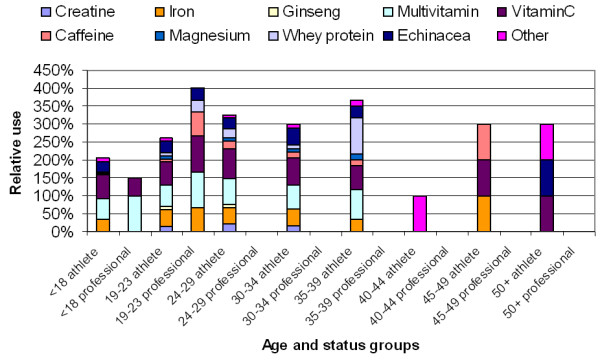
Relative use of nutritional supplements by age and status among female supplement users (n = 520).

Figures 3 and 4 show concomitant use of nutritional supplements by age and status groups for both genders. The use of each supplement itself represents 100% (which is the case if all supplement user athletes reported the use of a given supplement) and the reported usage of each supplement by age and status groups are stacked to facilitate comparison between the subgroups. From the graphs it is apparent that a fairly balanced multiple use of supplements is a characteristic of the male athlete supplement user population (Figure 3). Female supplement users reported taking fewer supplements in combinations and their choices appeared to be dominated by health maintenance with multivitamin and vitamin C (Figure 4).

**Figure 5:**
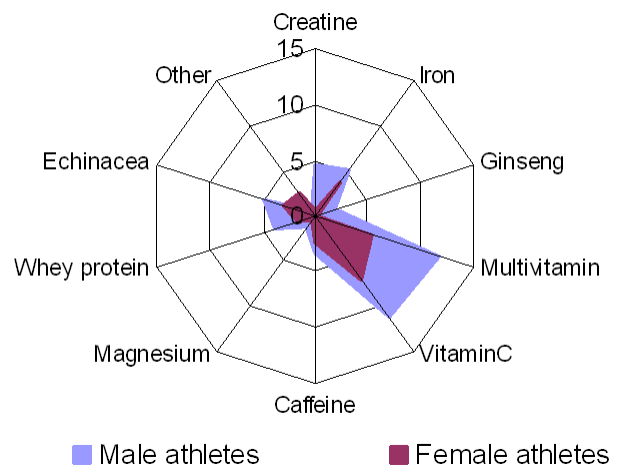
Relative percentage of each supplement used by gender (n = 520).

Multivitamins and vitamin C were equally the most ingested supplement by athletes from both genders (Figure 5) but the proportion of female users was less than the proportion of male users. Creatine was predominantly used by male athletes whereas the use of iron was relatively high among female athletes.

### Supplement use

The average number of supplements used by athletes in the users' sample was 3.22 (1674 instances with 520 athletes), indicating that supplements are used in combinations. Of the 520 supplement users, 82.6% reported the use of more than one supplement (Table 2). Among them, 23.7% used two and a further 21.6% used three supplements. This is congruent with the analysis of the declaration report used during drug testing at the 2000 Sydney Olympics [19], where alarmingly high overuses of vitamins were found among 2758 athletes. Concomitant use of nutritional supplements is typical in general population as well with a wider range of supplements ingested daily. Multiple supplement users tend to take a combination of multivitamin, B-complex, vitamin C, E, D, carotenoids, calcium, omega-3 fatty acids, flavonoids, lecithin, alfalfa, coenzyme Q10, glucosamine and herbal immune supplements daily [20]. In the UK sport sample, the list of nutritional supplements was limited to nine supplements plus the 'other' category. Even so, 11.5% reported using more than 5 supplements with dosage unknown. Higher than average (> 3.22) number of supplements used were reported in the following groups: 24–29 years old male athlete (4.03), 19–23 years old female professional (4.00), 29–29 years old male professional (3.84), 35–39 years old female athlete (3.67), 19–23 years old male professional (3.52), 35–39 years old male professional (3.47).

**Table 2:** Prevalence of multiple supplements use in the full sample (n = 874) and among supplement users (n = 520)

**Number of supplements reported**	**% of all athletes**	**% of all supplement users**
None	40.2	
1 supplement	10.4	17.4
2 supplements	14.2	23.7
3 supplements	12.9	21.6
4 supplements	9.8	16.4
5 supplements	5.6	9.4
6 supplements	3.2	5.4
7 supplements	1.8	3.1
8 supplements	1.0	1.7
9 supplements	0.8	1.3

## Conclusion

The results deserve attention for multiple reasons: i) nutritional supplements obtained from unknown sources may be contaminated and pose an involuntary doping offence [13], ii) a combination of supplements can potentially be dangerous [21], and iii) most importantly, supplement use increases the probability of prohibited substance use [2].

Assisting sports performance by allowable means is an acceptable behaviour to most athletes who are involved in competitive sport. Physiological, biomechanical, medical, psychological and nutritional supports are routinely provided to high performing athletes and during the natural course of the athlete development, athletes are accustomed to technologies and methods for human performance enhancement. According to the life-cycle model of performance enhancement doping practices likely to grow out of habitual engagement in a range of acceptable performance enhancement practices [22].

Therefore, the results of this paper have strong practical applications. Groups exhibiting high use of nutritional supplements should be given attention to prevent transgression toward prohibited methods. Research is urgently needed to identify risk factors, inform anti-doping deterrence and foster targeted interventions. As athletes are more likely to be honest about using supplements rather than reporting on prohibited substances, self-reported supplement use may be successfully applied as a proxy for the perceived need for assisted performance enhancement and is easily used as a screening tool. Future studies should incorporate a wide range of supplements and investigate the daily amount ingested. Groups exhibiting high use of nutritional supplements should be given attention to prevent transgression toward prohibited methods. Preventive measures should incorporate offering alternatives for assisted performance enhancement.

## Competing interests

The author(s) declare that they have no competing interests.

## Authors' contributions

AP sought out the data source, performed data analysis and drafted the manuscript. DPN assisted with data interpretation and contributed to the manuscript. All authors read and approved the final manuscript.
